# Cas9-Leveraged Single-Molecule Characterization of Sparse Plasmid Vectors in Heterogenous DNA Samples

**DOI:** 10.1007/s12010-025-05450-7

**Published:** 2025-11-08

**Authors:** Carl Möller, Luis Leal-Garza, Emanuele Celauro, Roberto Nitsch, Fredrik Westerlund

**Affiliations:** 1 Department of Life Sciences, Chalmers University of Technology, Gothenburg, SE 41296 Sweden; 2 Cell and Gene Therapy Safety, Clinical Pharmacology and Safety Sciences R&D, AstraZeneca, Gothenburg, Sweden

**Keywords:** Circular DNA, Cas9, YOYO-1, Single molecule, Fluorescence microscopy, Adeno associated virus (AAV), Episomal DNA, Concatemerization, DNA sizing

## Abstract

The detection and characterization of small circular DNA molecules, such as extrachromosomal circular DNA, non-viral episomal vectors, viruses, and plasmids, are crucial in biological and medical research. This study presents a method for identifying and sizing sparse populations of circular DNA in heterogeneous samples using single-molecule fluorescence microscopy. The method leverages the high sequence specificity of Cas9 combined with the fluorescence of YOYO-1-stained DNA to achieve single molecule sensitivity without the need for amplification or background DNA removal. The results show that a linear relationship between DNA size and fluorescence intensity can be achieved with simultaneous co-localization of Cas9 without significantly affecting size estimation. The method was applied to DNA samples from HEK293 cells transduced with recombinant Adeno-Associated Virus (rAAV) vectors. Characterization of DNA samples from the transduced cells revealed distinct subpopulations of viral vectors, highlighting the potential of the method for sensitive analysis of DNA molecules in complex and mass-limited samples. This approach provides a powerful tool for studying the mechanisms of rAAV vector transduction and maintenance, with broader applications in detecting and characterizing small circular DNA molecules in biological and medical research.

**Supplementary Information:**

The online version contains supplementary material available at 10.1007/s12010-025-05450-7.

## Introduction

Detection and characterization of small circular DNA molecules in heterogeneous samples is a recurring practice when investigating, for example, extrachromosomal circular DNA [1] (eccDNA), non-viral episomal vectors [2], viruses [3] and plasmids [4]. These circular DNA elements play crucial roles in a variety of biological processes. For instance, eccDNAs can carry oncogenes and contribute to drug resistance in cancer [5], while plasmids are central to horizontal gene transfer in bacteria, facilitating the spread of antibiotic resistance and metabolic traits [4]. Circular viral genomes, specifically recombinant Adeno-Associated Virus (rAAV), are known for their long-term episomal maintenance and low immunogenicity, making them suitable for treating genetic disorders [6].

Methods for detecting circular DNA include biochemical techniques such as rolling circle amplification (RCA), exonuclease digestion followed by PCR and Southern blotting [7]. Additional tools, such as electron microscopy [8] provide complementary insights into localization and abundance. Detecting and characterizing circular DNA is essential not only for understanding their functional roles in health and disease, but also for developing diagnostic tools and designing effective therapeutic strategies.

To size very small amounts of DNA, both capillary gel electrophoresis [9] and flow cytometry [10] are established techniques that can achieve picogram sensitivity. Capillary gel electrophoresis uses a gel matrix to partition molecules while flow cytometry relies on the relation between molecule size and fluorescent dye binding, measuring an intensity burst as the DNA molecule passes the detector [10, 11]. Using the same principle, and depositing DNA stained with a fluorescent dye on a functionalized glass surface, it is possible to obtain single-molecule resolution and reach femtogram sensitivity [12, 13]. While sizing of single DNA molecules deposited on a surface relies on population-based means for robust results, the method inherently produces detailed information for each molecule, such as intensity variation and shape descriptors. This feature has, for example, been successfully used to distinguish large plasmids containing different antimicrobial resistance genes [14].

A challenge when studying DNA in heterogeneous populations is generating a detectable signal with a sufficient signal-to-noise ratio (SNR). This often requires that the DNA of interest exists in a sufficient amount and/or that the sample has discrete, separate populations, to achieve robust differentiation. Samples can be preprocessed to reduce diversity into discernible populations [3, 15–17], either by amplifying the DNA of interest or removing background DNA. Amplification is beneficial when the DNA of interest exists in few copies and greatly enhances the SNR, but reduces information about concatemerization and repetitive sequences. Enzymatic removal of background DNA typically requires a large sample and prior knowledge of the DNA conformation so that the DNA of interest can be excluded from an enzymatic digestion, e.g. removal of linear DNA by exonucleases, or exists in a large enough quantity to be readily isolated.

Fluorescence In Situ Hybridization (FISH) has long been an effective method to detect and quantify specific sequences of DNA or RNA inside and outside of cells [18, 19]. Traditionally, fluorescently labeled single-stranded DNA probes have been used for hybridization to the target sequence, but this process involves lengthy and complicated protocols where the DNA must be fixated and denatured to ensure stable hybridization of the probe [19]. To circumvent the need for denaturation before hybridization, attempts have been made to develop enzymatic approaches. An early protocol used RecA to unwind DNA and hybridize an oligo to the target sequence [20]. In more recent efforts, researchers have turned to the CRISPR-Cas system to develop alternatives like CASFISH [21], RGEN-ISL [22] and CRISPR-FISHer [23]. These methods use a nuclease deficient Cas9 (commonly referred to as dead Cas9 or dCas9) modified with a fluorescent label either on the protein itself and/or on the guide-RNA (gRNA) for fluorescently tagged sequence specific binding to DNA.

Adeno-Associated Virus (AAV) vectors have been studied extensively over the past 50 years and have for more than half of that time been increasingly applied as a means of delivering genetic material to cells for therapeutic purposes [24, 25]. The use of recombinant AAV (rAAV) vectors for gene delivery typically involves cassettes containing the gene of interest coupled with regulatory elements flanked by inverted terminal repeats (ITRs). The ITRs are the only viral elements necessary for replication and packaging of the DNA into the viral capsid [26]. The DNA, which can be up to 4.7 kb long, is packaged in the viral capsid as single-stranded DNA (ssDNA), which upon entry into the host cell is converted to double-stranded DNA (dsDNA), either by annealing to a complementary strand or by synthesis primed by the ITR [6]. The transduced AAV vectors have been shown to primarily persist as episomal DNA in vivo, either in monomeric or concatemeric form [27], but can over time also integrate into the host genome [28]. Episomal persistence refers to the maintenance of the viral genome as an extrachromosomal element within the host cell nucleus. Unlike integration into the host genome, episomal DNA remains separate from host chromosomes. Episomal rAAV genomes are relatively stable and can persist in non-dividing cells for extended periods. The episomal persistence of AAV vectors has been shown both in vitro and in vivo [28] and studies have revealed that these vectors can remain functional over time in e.g. muscle tissue [27, 29]. Key factors for long-term stability and expression from episomal rAAV vectors include circularization and formation of concatemers [3, 29, 30]. To further understand the mechanisms behind transduction and maintenance of rAAV vectors it is important to effectively be able to map the state of transduced vectors at any given time point and titer. Understanding the mechanisms of concatemerization and episomal persistence can help optimize rAAV vector design and improving their efficacy and safety in clinical applications.

In this work, we aimed to develop a method that can identify sparse DNA molecules in a heterogeneous sample using picogram quantities of input material, without the need for amplification or removal of background DNA. The approach utilizes the high sequence specificity and affinity of dCas9 coupled with the linear dependence of YOYO-1 fluorescence intensity on DNA size, to identify DNA molecules and determine their size. From the colocalization of the YOYO-1-stained DNA and fluorescent dCas9-RNA complexes, DNA molecules of interest can be readily isolated from background DNA without preprocessing of the sample. To validate the method, we applied the protocol to DNA samples extracted from rAAV transduced HEK293 cells, and demonstrated that we could determine the size distribution of rAAV vectors residing in the cells.

## Methods & Materials

###  Cell Culture and Preparation 

Human embryonic kidney 293 (HEK293) cells were obtained from a certified cell bank and cultured in Dulbecco’s Modified Eagle Medium/Nutrient Mixture F-12 (DMEM/F12) supplemented with 10% fetal bovine serum (FBS). Cells were maintained in a humidified incubator at 37 °C with 5% CO₂. Prior to transduction, HEK293 cells were seeded in 96-well plates at a density of 1 × 10⁴ cells per well, allowing them to reach approximately 70–80% confluence at the time of infection.

### Viral Particle Preparation and Transduction

Adeno-associated viral (AAV) vectors containing the GFP-U6-gRNA-XM cassette were manufactured by Vector BioLabs. The viral titer was verified using quantitative PCR (qPCR) specific for the ITR sequences to ensure accurate multiplicity of infection (MOI) calculations. Transduction experiments were conducted by adding AAV-GFP-U6-gRNA-XM particles to the HEK293 cultures at varying MOIs: 0, 10, 100, 1000, and 10,000. These MOIs were selected to cover a wide range of viral exposure, from minimal to excessive, to assess the dose-dependent efficiency of transduction.

### Incubation, Monitoring and Analysis

Following the addition of viral particles, cells were incubated undisturbed at 37 °C to facilitate viral entry and gene expression, until 72 h post transduction. Transduction efficiency and GFP expression were monitored using the Incucyte Live-Cell Analysis System. Imaging was performed at 10x magnification using both phase contrast and GFP fluorescence channels. This enabled visualization and quantification of GFP expression, providing insights into the impact of different MOIs on gene delivery efficiency. Transduction efficiency was calculated as the summed GFP intensity relative to the cell population. The intensity of GFP fluorescence served as an indicator of expression levels across varying MOIs. Data were statistically analyzed with Dunnett’s multiple comparison test to determine which MOI resulted in GFP expression above the autofluorescence background.

### DNA Isolation

Total DNA extraction was done by resuspending cells in 300 µL lysis buffer (10mM Tris-HCl pH 8, 1 mM EDTA, 0.5% (w/v) SDS, 100 mM NaCl, 200 ug/ml Proteinase K and 100 ug/ml RNAse) and incubating the lysate at 65 °C for 30 min with shaking. The DNA was extracted with phenol: chloroform (UltraPure™ Phenol: Chloroform: Isoamyl Alcohol (25:24:1, v/v)) and precipitated with the addition of glycogen in ethanol.

### qPCR

Presence of rAAV vectors in the isolated DNA was confirmed and quantified by qPCR. For absolute copy number estimation, a standard curve was established using pAAV-U6-sgRNA-CMV-GFP (addgene: #85451) as a substrate. All reactions were performed with validated primers [31, 32] (**5’***GGAACCCCTAGTGATGGAGTT*’**3** and **5’***CGGCCTCAGTGAGCGA*’**3**) and PowerTrack™ SYBR™ Green Master Mix (applied biosystems) on an Agilent Stratagene Mx3005P system with ROX as a reference dye. Setup and cycling were performed according to the manufacturers protocol.

### Cas9 in Vitro Digestion and EMSA

All in vitro digestion experiments were done with *S. pyogenes* Cas9 (NEB M0386S) in NEBuffer r3.1, if nothing else was specified. Electromobility shift assay (EMSA) was performed with dCas9 (NEB EnGen^®^ Spy dCas9 (SNAP-tag^®^)) in NEBuffer r3.1 adjusted to 1 mM MgCl_2_. All reactions were analyzed with gel electrophoresis in 1x TAE 1% agarose gels run at 80 V for 120 min. All RNA was purchased from IDT.

### Standard Reference Sample Preparation

The following plasmids were used for establishing a size-intensity standard: pUC18 (addgene: #50004), pBR322-TIMER (addgene: #103056) and pGreenIIM RPS5A-mDII-ntdTomato/RPS5A-DII-n3Venus (addgene: #61629). The DNA samples were labeled with a 1:1 YOYO-1 to basepair(bp) ratio by adding 1 ng of DNA to 10 µL buffer (10 mM TRIS-HCL, 1 mM EDTA, 200 mM DTT) with 154 nM YOYO-1. The labeling reaction was incubated overnight at 50 °C.

### Fluorescent Cas9 Co-Localization

For sequence specific identification of DNA molecules, a dCas9 (NEB EnGen^®^ Spy dCas9 (SNAP-tag^®^)) coupled with a fluorescently labeled tracr-RNA (Alt-R CRISPR-Cas9 tracrRNA - ATTO™ 550) and an AAV ITR-targeting crRNA (**5’***AGUGGCCAACUCCAUCACUA***’3**) was used. The cr: tracrRNA was annealed by mixing a 1:1 ratio (10 µM) in IDT Duplex Buffer (30 mM HEPES, pH 7.5; 100 mM potassium acetate) heated to 95 °C for 5 min and allowed to reach room temperature. The dCas9 and RNA were complexed by mixing equimolar amounts of cr: tracrRNA and dCas9 in NEBuffer r3.1 (1 mM MgCl_2_), incubating for 10 min at 37 °C and diluting to 10 nM. The diluted dCas9-RNA complex was kept on ice until use. The reaction was set up by mixing 1 ng of DNA in 9 µL NEBuffer r3.1 (1 mM MgCl_2_, 17 nM YOYO-1). 1 uL of dCas9-RNA (10nM) was added and incubated for 15 min at 37 °C before 8 µL was loaded on functionalized glass. All RNA was purchased from IDT.

### Glass Functionalization

Glass coverslips were functionalized according to published protocols [14, 33–36]. In short, coverslips (Epredia 22 × 22 mm #1.5) and microscope slides (Epredia Plain Cut 25 × 75 mm) were first washed by sonication in a 2% Hellmanex III solution for 30 min and rinsing 3 times with MilliQ (MQ) water. The microscope slides were kept in MQ water until imaging. The coverslips were dried with N_2_-gas and rinsed with acetone to remove any residual water. The coverslips were then silanized by incubating them in a mixture of Allyltrimethoxysilane (ATMS, 95%, Sigma-Aldrich), (3-aminopropyl)triethoxysilane (APTES, ≥ 98%, Sigma-Aldrich) and Acetone (Sigma-Aldrich) in a 1:1:100 ratio for at least 2 h at room temperature. Prior to loading a sample, both the coverslip and microscope slide were quickly rinsed with MQ water and dried with nitrogen. An 8 µL drop of sample was sandwiched between a functionalized coverslip and a microscope slide and immediately imaged.

### Microscopy

Imaging was done with an inverted fluorescence microscope (Zeiss AxioObserver.Z1) equipped with a 63x oil immersion objective (NA = 1.46, Zeiss) coupled with a 1.6×optovar magnification and an iXon EMCCD camera (Andor). For illumination an LDI-7 Laser Diode Illuminator (89 NORTH) was used. Sample was excited with 555 nm or 470 nm light and filtered with DsRed or 44 FITC filters (Zeiss), respectively. Images taken in the ATTO550 channel were exposed for 500 ms with 200 EM gain and images in the YOYO-1 channel for 300 ms and with 50 EM gain. Images were collected in 10 × 10 grids with adjustment of the focus via fluorescently guided autofocus. The YOYO-1 channel was used to set the focus reference.

### Image Analysis

Images were processed using in-house Python scripts that, in short, were based on a QC pre-processing step and a segmentation step. See Supplementary Fig. 1 for a complete outline. The QC consisted of a manual inspection where images that were out of focus or contained artifacts were discarded. The preprocessing step involved shading correction to mitigate uneven illumination and filters to remove noise, see Supplementary Fig. 2 for a representative example. The segmentation step involved thresholding by an edge finding algorithm and a local thresholding algorithm to account for local variations. The resulting masks were then merged and used to locate and outline DNA puncta, Supplementary Fig. 3. The extracted intensity data could then be used to generate histograms from which each individual population could be identified and quantified, see Supplementary Fig. 4 for a detailed description.

## Results

The aim of this work was to develop and validate a method for detection and size determination of sparse circular DNA in heterogeneous samples using single-molecule fluorescence microscopy. The method is based on two established principles - the relationship between the molecular weight of a DNA molecule and the emission from bound YOYO-1, and the stable sequence specific binding of dCas9 to DNA. The dCas9 was fluorescently labeled via the tracrRNA (Alt-R CRISPR-Cas9 tracrRNA - ATTO™ 550). To test the applicability and sensitivity of the method, HEK293 cells were transduced with rAAV with the aim to identify and characterize the size distribution of the expressed viral vectors post transduction.

### Fluorescence Intensity Scales Linearly with DNA Size

The linear dependence between fluorescence intensity of intercalated dyes and DNA size is well established [11, 12, 37]. A mix of three plasmids (2686 bp, 5562 bp and 12920 bp) was used to generate a calibration curve to map the size-intensity relation for circular DNA. Each reference plasmid was stained separately with a 1:1 bp:YOYO-1 ratio by incubating 1 ng DNA in 10 uL TE with 200 mM DTT and 154 nM YOYO-1 at 50 °C overnight. The individual plasmid preparations were then mixed to form a heterogeneous sample with three discrete populations. Of this mixture, 800 pg of DNA was deposited on functionalized glass slides and imaged.

**Figure. 1:**
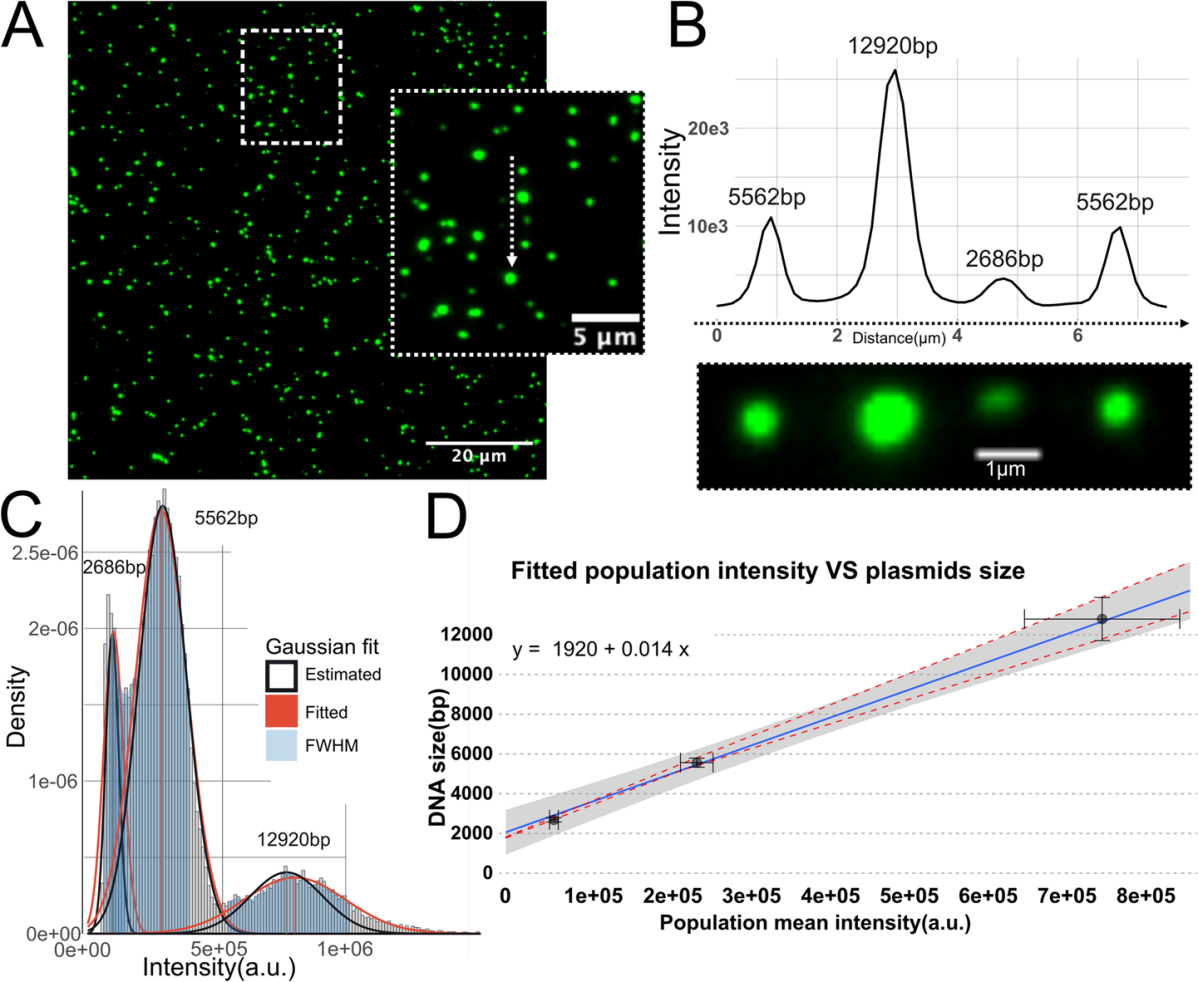
**A** Representative raw image data from a mixed plasmid sample with 2686 bp, 5562 bp and 12,920 bp plasmids stained with a 1:1 bp: YOYO-1 ratio and deposited on functionalized glass. **B** Intensity profile along the particles indicated by the white dashed arrow in the inset of A along a close-up of the quantified particles. **C** Histogram of molecule intensities with fitted gaussians from a representative experiment with a mixed plasmid sample. Each molecule intensity is background adjusted. **D** Regression line fitted to known DNA size vs. molecule intensity mapping based on the average population intensity extracted from fitted gaussians with the 99% CI outlined in gray. Horizontal error bars represent the SD of the population means from four replicates thus representing the deviation in average population intensity between experiments while the vertical error bars represent the average estimation error with the red dashed line outlining the upper and lower bounds of the estimation error along the regression

Figure 1A shows a representative image from such an experiment together with an inset with examples of the three plasmids. Figure 1B shows the intensity profile of the individual molecules marked in the inset in Fig. 1A together with a zoomed and cropped copy of the inset to further illustrate how the three plasmids can be readily separated. The YOYO-1 intensity scales linearly with DNA size and there is a similar intensity for plasmids of the same size, as is demonstrated by the examples in Fig. 1B.

By plotting the distribution of the molecule intensities, a population mean of each reference DNA size could be determined (Fig. 1C). This was done by approximating the position of each peak, calculating the full width half maximum (FWHM), and estimating an initial Gaussian distribution. A Gaussian was then fitted to the subset defined by the initial estimate (see Supplementary material for a full description), whereafter a linear regression was performed on the population means and the known DNA size to get an expression that mapped intensity to DNA size, with an R^2^ = 0.99 (Fig. 1D**)**. If the regression was coerced through origo, the accuracy of the fit decreased to R^2^ = 0.97, which is a marginal decrease, but based on the residuals this would result in an underestimation of all sizes. Importantly, the variance of the population means increases with increased peak width. It has previously been reported that the estimation error indeed increases with larger DNA size, but that the relative error (height to width ratio) decreases [11, 12]. This is also true for the data reported in Fig. 1C.

### EMSA Shows That dCas9 Binds DNA in the Presence of YOYO-1

Since the specificity of a Cas9-based assay depends on the sequence of the guide RNA used to target the DNA molecule of interest, initial experiments explored several guide sequences to identify the most efficient one. Since parts of the sequence of the transduced vector were known, three guide sequences targeting the GFP gene, three targeting the AAV2-ITR, and one targeting the pBR322-ori were designed and tested for digestion efficiency using gel electrophoresis (see Supplementary Table 1 for all RNA sequences and data). Supplementary Fig. 5 shows representative results from the reaction. From this assay it was concluded that the most efficient guide RNA targeted the AAV2-ITR (5’ AGTGGCCAACTCCATCACTA) with 92+/−2% of the available DNA being linearized.

Previous findings have demonstrated that Mg^2+^ can interfere with YOYO-1 binding to DNA [38, 39]. To mitigate this, we wanted to minimize the MgCl_2_ concentration while still maintaining Cas9 DNA-binding. Additionally, it has been shown that YOYO-1 can interfere with the activity of exonucleases [40]. To address this, the bp: dye ratio and order of addition was optimized for proper dCas9 activity. The results show that 1 mM MgCl_2_ is sufficient for proper activity, but a bp: dye ratio above 10:1 (bp: dye) is inhibitory. The order of addition is inconsequential for dCas9 activity, but has an apparent effect on the final fluorescence signal. See Supplementary "Guide design optimization" and Supplementary Fig. 6A-D for a comprehensive outline of these experiments and results.

### dCas9 Bound to YOYO-1 Labeled Plasmid DNA Does not Affect Size Estimation

**Figure. 2:**
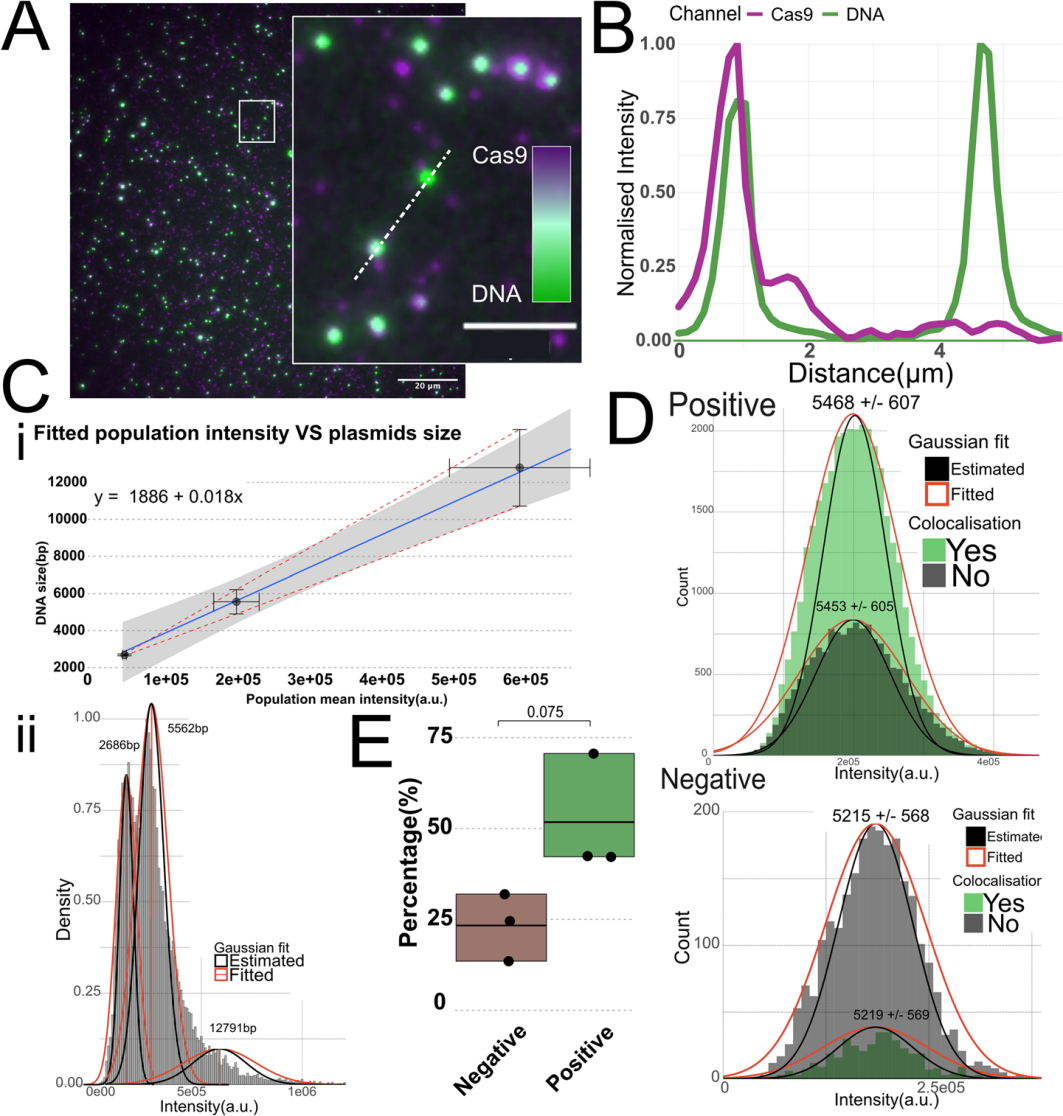
**A** Representative image of colocalization between plasmid DNA (pAAV-U6-sgRNA-CMV-GFP) and dCas9 complexed with ATTO550-labeled RNA targeting the AAV2-ITR. **B** The 2D profile shows the normalized intensity of each fluorescent channel across the pixels indicated by the white dashed line in the inset of **A**. **Ci** Regression line fitted to known DNA size vs. molecule intensity imaged under dCas9 binding conditions. Points are based on the average population intensity extracted from fitted gaussians with the 99% CI outlined in gray. Horizontal error bars represent the SD of the population means from three replicates thus representing the deviation in average population intensity between experiments while the vertical error bars represent the average estimation error with the red dashed line outlining the upper and lower bounds of the estimation error along the regression. **Cii** Histogram of molecule intensities with fitted gaussians from a representative experiment with a mixed plasmid sample. Each molecule intensity is background adjusted. **D** Histogram of detected DNA particles from experiments including plasmid DNA with (Positive) and without (Negative) complementary sequence for AAV2-ITR gRNA. Particles are categorized based on colocalization and the histograms from each category are overlaid. The population average intensity is converted to base pairs with the equation reported in Fig. 2C and the estimation given as a +/- range. **E** Relative colocalization degree for multiple experiments with positive and negative plasmids

To determine if the AAV2-ITR targeting guide RNA was binding equally well in the single-molecule assay, the pAAV-U6-sgRNA-CMV-GFP vector (addgene #85451) was used as a proxy substrate. Figure 2A shows a representative image from an experiment with YOYO-1 labeled DNA and dCas9-RNA complex conjugated with a ATTO550 fluorophore. The DNA can be identified as green puncta and ATTO550 fluorophores as magenta puncta. The inset highlights multiple examples where the green and magenta puncta co-localize, indicating that dCas9 is bound to DNA. Figure 2B shows the intensity profile along the dashed line in the inset in Fig. 2A which highlights a co-localization event and shows in detail the spatial overlap of the intensity from an ATTO550 fluorophore and a DNA punctum.

Since the buffer conditions and YOYO-1 concentration used in the dCas9 experiments differed significantly from those used for the calibration curve in Fig. 1D, a new calibration curve was established using the same plasmid sizes, but under the dCas9 buffer conditions (Fig. 2C**)**. Figure 2Ci shows the resulting regression performed on the population means and the known DNA size. Figure 2Cii depicts the distribution of molecule intensities for the data used in Fig. 2Ci. To determine if the sizing standard was valid with co-localization and benchmark the dCas9 co-localization, experiments with two different plasmids were compared. One plasmid containing the target sequence, pAAV-U6-sgRNA-CMV-GFP (5181 bp), and one plasmid of similar size without the target sequence (pBR322, 5562 bp). Fig. 2D shows the size distribution for all detected particles categorized as co-localized overlaid with the distribution of molecules determined to be non-colocalized. A DNA molecule was categorized as co-localized if there was a sufficiently high correlation between the intensity of the DNA channel and the same pixels of the dCas9 channel. The threshold was set to PCC > 0.25 with a p-value < 0.05 ( Supplementary “colocalization statistics” for further details).

The mean of the fitted Gaussian from each population provides the intensity value, from which the size can be extrapolated using the expression in Fig. 2C. In the included example (Fig. 2D) the sizing of the positive control plasmid is overestimated by 284 bp (+ 5.5%), while the negative control is underestimated by 347 bp (−6.2%), although the true size lies well within the estimation error in both cases. The alignment between the histograms of the co-localized and the non-colocalized particles confirms that the binding of dCas9 to the DNA does not skew the size estimation outside of the error range. Figure 2E reports the average relative co-localization degree for DNA with (51%) and without (23%) a dCas9 target site and shows that there is a non-negligible degree of unspecific co-localization. A student’s t-test suggests that the difference between conditions is not significant but with a p-value = 0.075. This background co-localization could be due to unspecific DNA-binding by dCas9, as observed in bulk experiments, or because of the random deposition on the surface. Since proper binding of dCas9 to DNA requires a 10:1 stoichiometry, there is an excess of dCas9 added to the reaction. This inevitably increases the risk of having false-positive colocalization. Since the colocalization was based on the detection of a single fluorophore per dCas9. one concern was false-negatives due to low labeling efficiency of the RNA. The labeling degree was determined spectrophotometrically to be 88 ± 2% (Supplementary Fig. 7, Supplementary Table 2), indicating that the effect of unlabeled RNAs was limited, but not negligible.

### GFP expression Confirms the Presence of rAAV in Transduced Cells

**Fig. 3:**
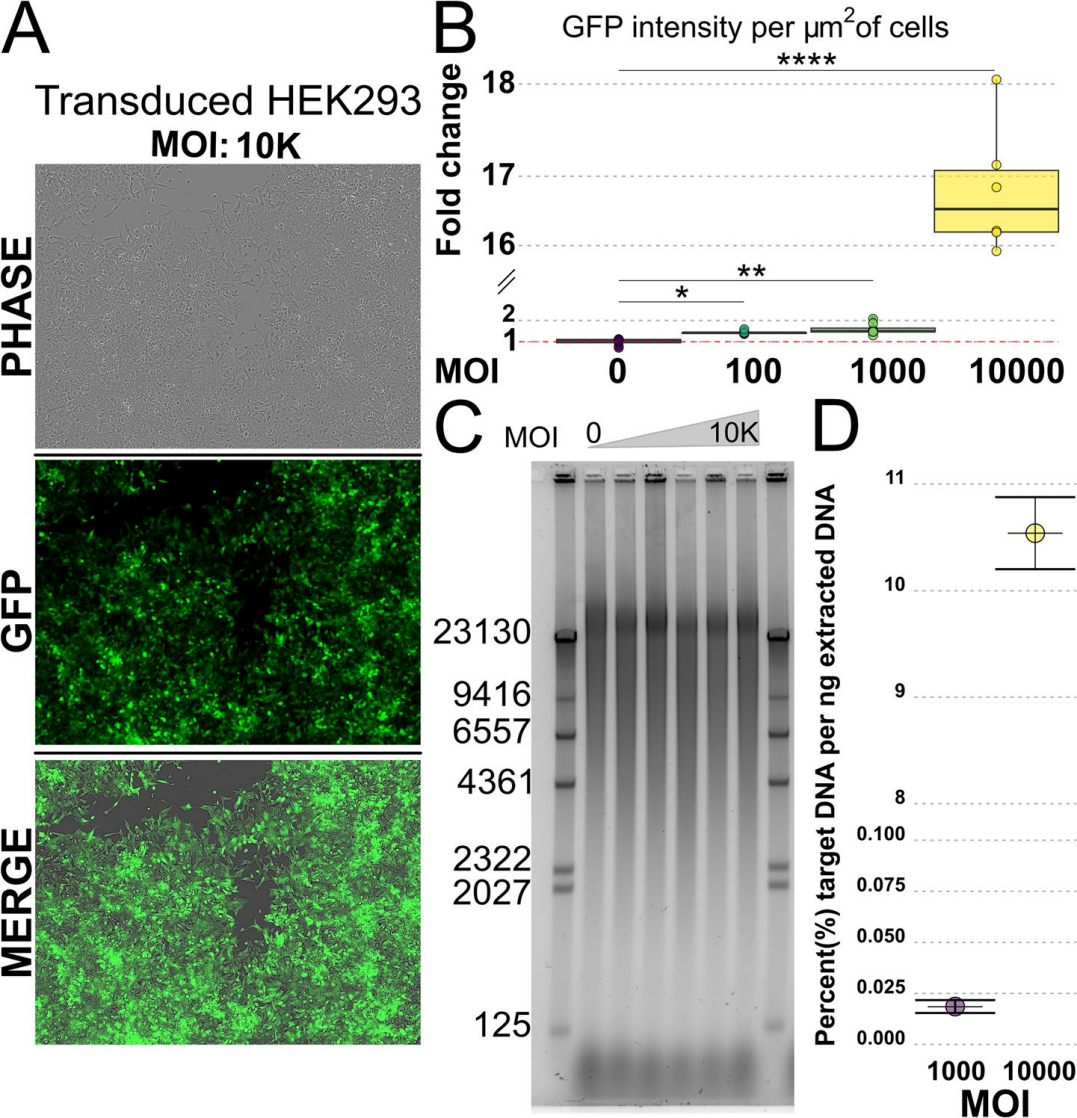
**A** Representative image of HEK293 cells transduced with MOI 10 K AAV-DJ8 virus expressing GFP at 72 h after transduction. The image set includes phase contrast, FITC and a merged image. **B** Quantification of GFP intensity in HEK293 cells based on the fluorescent intensity per area unit of cells reported as a fold change relative to the MOI 0 condition. Statistics were performed with Dunnett’s multiple comparisons test. Only significant conditions are displayed. **C** Results from electrophoresis of DNA extracted from HEK293 cells transduced with rAAV at various MOI. **D** Relative mass of viral vectors per nanogram of extracted DNA based on copy numbers found by qPCR data. No conditions below MOI 1 K showed signal above background and are therefore excluded. See **Supplementary Fig. 8A** for further details

To validate the protocol on a heterogeneous sample with a subpopulation of target DNA, HEK293 cells were transduced with AAVDJ8 with a step gradient of MOI ranging from 1 to 10,000. The viral vector was loaded with a GFP expression cassette. Figure 3A shows a representative image of the transduced cells from the highest MOI used, where the GFP signal is visible in all cells. The GFP expression was quantified by segmenting the phase image to exclude areas without cells and normalizing the integrated GFP intensity against the area of the cells. Figure 3B reports the results from this quantification as a fold-change compared to the MOI 0 condition. A Dunnett’s multiple comparisons test concluded that only MOI 100 (*p* = 0.048), 1 K (*p* = 0.004) and 10 K (p < < 0.0001) had a GFP-signal significantly higher than the autofluorescence background of the MOI 0 sample. The transduced cells were subsequently lysed and the DNA was extracted with phenol: chloroform and ethanol precipitation. To check the quality of the extracted DNA and to get an overview of the size distribution, the extracted DNA was analyzed with gel electrophoresis (Fig. 3C). The results indicate that the target DNA is present at levels too low to be distinguished from the background cellular DNA. Additionally, the gel reveals that the extracted DNA from all samples contains DNA fragments ranging from 125 bp to more than 23 kbp in size.

### qPCR Based Estimation of Viral Vector Copy Number in Cell-Derived DNA Samples

The quantification of the expressed GFP confirmed that the transduced cells retained at least the parts of the viral vector necessary for GFP expression. Since AAV vectors are known to integrate into the host genome, the GFP expression in itself did not confirm that the cells maintained any episomal viral vectors. To further confirm the presence of viral vectors and estimate the actual copy number that could be expected in each sample, qPCR was performed with primers targeting the AAV-ITR2 region. A calibration curve to map the initial copy number to the cycle threshold (Ct) was established using pAAV-U6-sgRNA-CMV-GFP as a proxy. The curve was constructed from six points, with copy numbers ranging from 80 to 8 × 10^6^ plasmids. Due to the non-template-control (NTC) generating a Ct value below the average of the 80-8 × 10^3^ copy number condition in the calibration curve and that of MOI 0–100, it was taken as the true background signal to define the limit of detection (LoD). Conditions with a Ct value below the LoD were omitted in the subsequent analysis. Using the calibration curve, the initial copy number for each condition was estimated, which gave a first indication of how many viral vectors each cell-derived DNA sample contained. The copy number was then converted to mass under the assumption that each copy equated to a plasmid with a size of 2684 bp and normalized to the amount of ingoing DNA in the qPCR reaction. This suggested that the relative mass of viral vectors per nanogram of extracted cellular DNA for the MOI 10 K condition to be around 10.5% while only 0.025% of the extracted DNA from the MOI 1 K condition could be expected to be viral vectors (Fig. 3D). Using the estimated relative mass suggests that 800 pg of extracted DNA from MOI 10 K (mass used for imaging) contains around 3 × 10^6^ copies of monomeric viral vectors, which can be equated to 1 viral vector per 15µm^2^ or a theoretical maximum of ~ 1000 viral vectors per image. Doing the same calculations for MOI 1 K gives a density of 1 viral vector per 8950 µm^2^ which equates to a theoretical maximum of < 2 vectors per image. From these estimations and the GFP quantitation it was apparent that MOI 1 K did not contain a sufficient number of vectors for further analysis. MOI 10 K was the only condition included in the subsequent analysis. The full qPCR results and regressions are reported in Supplementary Fig.  8A-D. It is important to note that the cell-derived DNA samples contained both episomal and genomic DNA which entails that the results will include rAAV vectors integrated into the host genome.

### Intensity-Based Sizing Identifies Populations of Viral Vectors in Cell-Derived DNA Samples

**Fig. 4:**
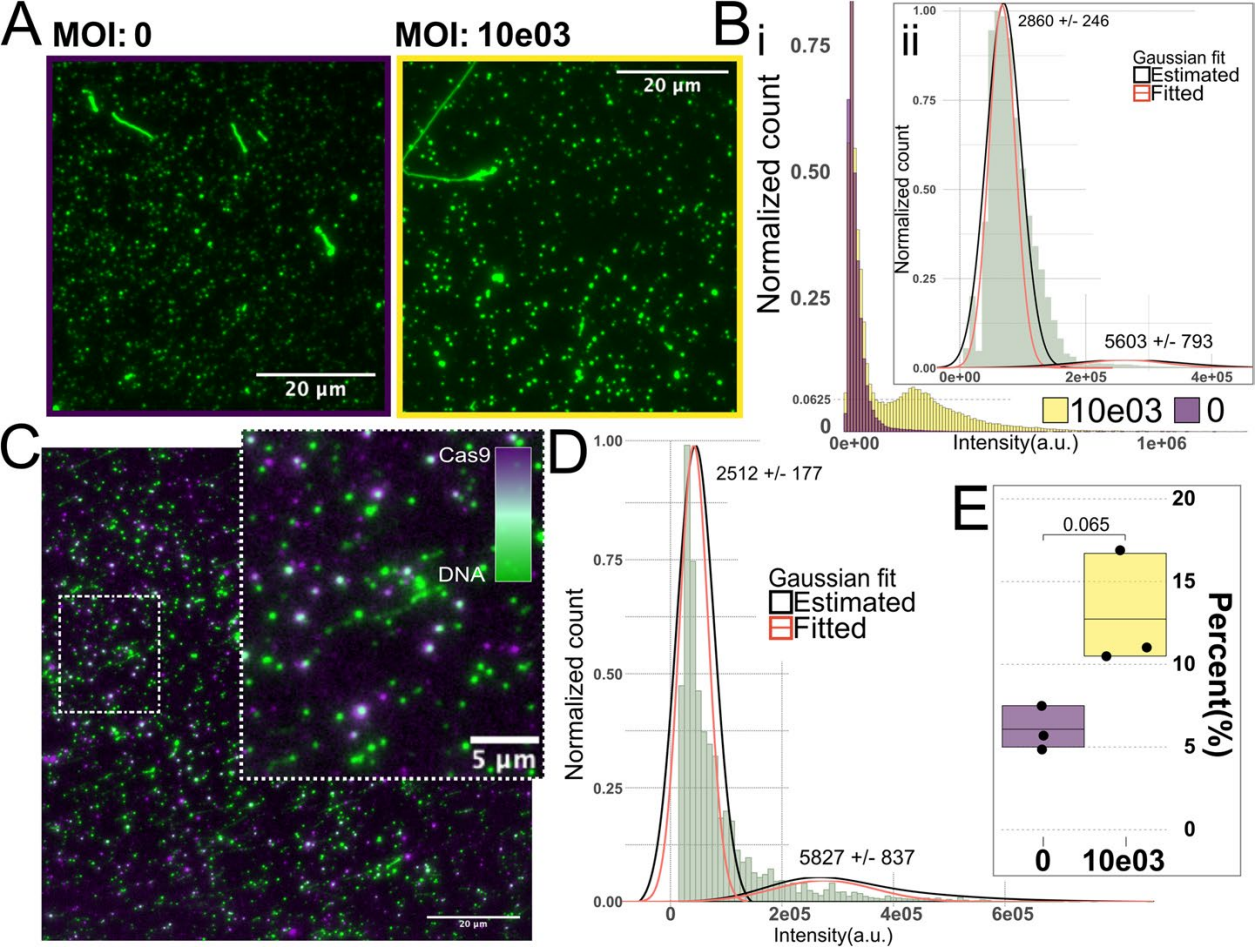
**A** Representative images of DNA extracted from cells, stained at a 1:1 bp: YOYO-1 ratio. The image to the left, outlined in purple, shows DNA from a negative control. The image to the right, outlined in yellow, shows DNA from cells transduced with MOI 10 K. **Bi** Overlaid histograms showing the distribution of molecule intensities from a representative DNA sample from cells transduced with MOI 10 K rAAV and a negative control sample. Bii inset shows the molecule intensity histogram of pooled data from multiple MOI 10 K samples after subtraction of the MOI 0 population together with fitted gaussians. The size of each population is stated with its estimation error and was extrapolated from the equation given in Fig. 1D. **C** Representative image of colocalization between cell derived DNA (MOI 10 K) and dCas9 complexed with ATTO550-labeled RNA targeting the AAV2-ITR. **D** Histogram of the background adjusted colocalized fraction of detected DNA in MOI 10 K based on pooled data from multiple experiments. 1 d K-means clustering was used to identify individual populations and the base pair size of each population was given by the equation from Fig. 2**Ci** together with the associated estimation error. **E** Relative colocalization degree from multiple experiments using cell derived DNA from either MOI 0 or MOI 10 K

From the gel electrophoresis reported in Fig. 3C, all conditions show a similar size distribution and there is no discernible difference between the MOI 0 and 10 K conditions. Figure 4 A shows fluorescence images for one MOI 0 sample and one MOI 10 K sample stained at a 1:1 bp:YOYO-1 ratio. When comparing the distribution of molecule intensities, it is apparent that the MOI 10 K sample includes subpopulations that are not present in the MOI 0 condition, as well as overlapping subpopulations (Fig. 4B). By assuming that the MOI 0 sample represents the background generated by genomic DNA and subtracting the MOI 0 histogram from the MOI 10 K histogram the difference would represent subpopulations present in the MOI 10 K condition that are not present in MOI 0. Figure 4Bi reveals this difference when overlaying the distribution of molecule intensities from MOI 0 and MOI 10 K. The histogram includes the molecule intensity distribution from one replicate and provides a *best-case scenario* that includes very defined populations at the monomer (~ 50e05 a.u.) and dimer (~ 250e05 a.u.) intensity with a tail extending into the intensity mean of a putative trimer (500e05 a.u.). This example demonstrates that the method can have the resolving power to distinguish the concatemeric populations of the used rAAV vector. The distribution Fig. 4 Bi is not true for all replicates and the inset (Fig. 4B**ii**) shows the histogram of background subtracted data from several experiments and provides a more representative view on the size and relative abundance of rAAV vectors. This *difference histogram* (4Bii) still reveals subpopulations around 2869 bp and 5603 bp, which are in line with the expected sizes of monomeric (2684 bp) and dimeric (5368 bp) rAAV vectors. Comparing the relative number of molecules included in each subpopulation shows that the monomeric fraction makes up the vast majority (98%) of the rAAV DNA, while the dimeric fraction only makes up 2%. After background subtraction, on average ~ 7% of the analyzed molecules remain. Assuming all the molecules in the background-subtracted data are rAAV vectors this would be in line with the ~ 10% estimate that was extrapolated from qPCR data (Fig. 3D).

### dCas9 can be Used to Confidently Resolve Sparse DNA Populations in Heterogenous Samples

To confirm that that the subpopulations detected by single-molecule sizing indeed were the rAAV vectors of interest, we added fluorescently tagged dCas9 to 1 ng of YOYO-1-stained DNA. Afterwards, 800 pg of the dCas9 bound DNA was deposited on functionalized glass and imaged. Again, we compared DNA from the MOI 0 and MOI 10 K conditions and used the results from MOI 0 for background subtraction. Figure 4 C provides a representative example from such an experiment and the inset shows that, despite adjacent molecules having similar intensities, some are not colocalized with a dCas9 and are thus most likely not an rAAV vector. Figure 4D shows a histogram of molecule intensities from only dCas9 co-localized molecules. The histogram consists of background subtracted data from multiple experiments. The identified subpopulations are similarly interspersed with a main population at 2512 bp that is taken to represent a monomeric rAAV vector (2684 bp) and a subpopulation at 5827 bp, around the size of the dimer (~ 5368 bp). Although molecules with intensities around a putative trimer are present, there are not enough of them to suggest the existence of a true subpopulation. When comparing the relative abundance between the monomeric and dimeric subpopulations, the monomeric subpopulation is present as a majority (88%). The subpopulation taken to be dimeric rAAV vectors makes out 12% of the total number of molecules which is 10% more than the dimeric subpopulation indicated by experiments without dCas9 (Fig. 4Bii). Comparing the average colocalization degree between MOI 0 (6.0%) and MOI 10 K (12.8%), Fig. 4E, revealed that there was a non-negligible amount of co-localization in the MOI 0 samples. A students t-test shows that the difference between conditions was not significant, but with a p-value = 0.065. Interestingly, the proportional co-localization degree between positive and negative samples is the same for cell-derived DNA as for plasmid DNA (Fig. 2E), i.e. the degree of co-localization in negative samples is roughly 50% of the co-localization in positive samples.

## Discussion

In this study, we have successfully developed a single-molecule method for sizing sparse populations of circular DNA in heterogeneous samples without the need for amplification or enzymatic preprocessing of the sample. We do this by coupling the co-localization of a fluorescently tagged dCas9-RNA with intensity-based DNA sizing. This method is especially suited for low mass samples since the assay utilizes less than 1 ng of DNA to perform a full analysis. This equates to roughly 150 cells, but can potentially be minimized to the single cell level.

In the development of the method, we found that the addition of dCas9 to the intensity-based sizing protocol required a different buffer composition and bp:dye ratio compared to the protocol initially used. Therefore, we established a second size: intensity calibration curve to be used in experiments that included dCas9. The difference between the two calibration curves was small and we observed that the size:intensity relation scaled similarly between the experimental conditions. The major difference was that when applying the first bp:dye ratio curve (1:1) on samples imaged in dCas9 buffer with bp:dye ratio of 9:1 the size of the DNA was underestimated by ~ 1000 bp on average, underscoring the importance of having separate calibration curves for each condition. Additionally, experiments demonstrated that dCas9 binds efficiently to target DNA sequences without significantly interfering with already bound YOYO-1. This finding is essential for the reliability of the method, as it ensures that the presence of dCas9 does not skew the intensity-based size estimations and show that the size: intensity relation of DNA and YOYO-1 is robust enough to be used in combination with additional DNA-binding partners.

To validate the method, we applied the protocol to DNA samples extracted from rAAV transduced HEK293 cells and determined the size distribution of rAAV vectors residing in the cells. The cell-derived DNA was characterized both with and without dCas9. In the analysis without dCas9 two major subpopulations could be identified that aligned with the anticipated sizes from monomeric and dimeric rAAV vectors. The findings are in line with previous reports [3, 16, 41] where such products have been extracted and isolated from tissues, although after a significant amount of time (6–12 months). While the results without dCas9 could identify the presence of multimers, reports have shown that the circularization and stabilization of transduced rAAV vectors can occur via recombination between the ITRs of panhandled fragments generating fractional combinations [42], which was not visible in the above-mentioned results. By using dCas9 to identify exactly which molecules had the ITR sequence, we were able to identify and resolve the size distribution of the rAAV vectors more definitively. These results demonstrated that the relative abundance of dimeric rAAV vectors was somewhat underestimated in data from experiments without dCas9. This application highlights the potential of the method for sequence-specific detection and characterization of sparse DNA molecules in low-mass biological samples and the use when studying various biological processes involving small circular DNA and to contribute in the development of gene therapy and other biomedical applications.

While the presented method shows great promise, there are some limitations to consider. The results from the EMSA experiments suggest that there is some degree of non-specific binding by dCas9. This was further evident in the single-molecule assay where a non-negligible degree of co-localization was detected in samples without a dCas9 binding site. This background can be in part explained by a small degree of false-positive co-localization due to dCas9 constantly searching for a complementary sequence. Since the assay is dependent on a single fluorophore per dCas9-RNA complex this creates an irreversible signal loss if the fluorophore is bleached or lost. Since the dCas9 binds tightly to its target, there will be no replacement of fresh fluorophores at these sites, in comparison to the transient binding during target search. This results in there being a fraction of true positives that are not detectable, while the fraction of false positives due to transient binding can be considered constant. Essentially this would result in an overestimation of false positives and an underestimation of the true positives.

Single-molecule fluorescence microscopy offers several advantages when analyzing sparse DNA populations. While bulk methods often require amplification or extensive preprocessing, single-molecule approaches provide direct, molecule-by-molecule resolution. This avoids biases that can be introduced from an amplification process and enables the characterization of structural heterogeneity, such as concatemerization. The ability to visualize individual DNA molecules also allows for the extraction of quantitative descriptors, such as fluorescence intensity, shape, and spatial localization. In this study, this capability was critical for distinguishing monomeric and dimeric forms of rAAV vectors and for validating sequence-specific binding using dCas9. Furthermore, the low input requirement (< 1 ng of DNA) makes the method compatible with limited or precious biological samples.

Despite its high sensitivity and resolution, single-molecule fluorescence microscopy has some inherent limitations. Data analysis can be computationally intensive and require robust algorithms for spot detection, intensity quantification, and co-localization. Additionally, it requires optimization of dye concentration, buffer conditions, and surface chemistry to minimize background noise and optimize adsorption. The reliance on surface immobilization also introduces the risk of DNA molecules adopting conformations or orientations that affect fluorescence intensity and the apparent size. This can however be controlled for by considering a large number of molecules.

## Conclusion

In conclusion, this study presents a single-molecule fluorescence microscopy method capable of detecting and sizing sparse populations of circular DNA in heterogeneous samples without the need for amplification or enzymatic preprocessing. By leveraging the high sequence specificity of dCas9 and the size to intensity relation of YOYO-1-stained DNA, this method offers significant advantages over traditional techniques.

The method was validated using DNA extracted from rAAV-transduced HEK293 cells and its ability to resolve monomeric and concatemeric forms of episomal viral vectors was demonstrated. Importantly, the addition of dCas9 enhanced the specificity of detection and revealed subpopulations that were underestimated by intensity-based sizing alone. These findings underscore the potential for studying circular DNA elements in low-mass biological samples, including those relevant to gene therapy, viral vector design, and the broader field of extrachromosomal DNA biology.

## Supplementary Information

Below is the link to the electronic supplementary material.

Supplementary Material 1 (DOCX 30.0 MB)

## Data Availability

The imaging data and analysis routines used for molecule detection are available and can be obtained by making a reasonable request to the corresponding author.
