# Supplementary material for: Cas9-Leveraged Single-Molecule Characterization of Sparse Plasmid Vectors in Heterogenous DNA Samples
Source: Appl Biochem Biotechnol. 2025 Nov 8;198(1):415–33. doi: 10.1007/s12010-025-05450-7 (PMC12894433; doi:10.1007/s12010-025-05450-7)
Supplement: Supplementary file 1 — Supplementary Material 1 (DOCX 30.0 MB) [file 12010_2025_5450_MOESM1_ESM.docx]

**Supplementary Information**

**Cas9 leveraged single molecule characterization of sparse plasmid vectors in heterogenous DNA samples**

Carl Möller*^a^*, Luis Leal Garza*^a^*, Emanuele Celauro*^b^*, Roberto Nitsch*^b^*, Fredrik Westerlund*^a*^*

*a. Department of Life Sciences, Chalmers University of Technology, Gothenburg, SE, 41296, Sweden*

*b.* *Cell and Gene therapy Safety, Clinical Pharmacology and Safety Sciences R&D, AstraZeneca, Gothenburg, Sweden*

* Corresponding author

E-mail address: fredrik.westerlund@chalmers.se (F. Westerlund)

Table of Contents

[1. Image and data processing 2](#_Toc187156412)

[Pre-processing 2](#_Toc187156413)

[Segmentation 3](#_Toc187156414)

[Co-localisation statistics 4](#_Toc187156415)

[Peak identification and Gaussian estimation 5](#_Toc187156416)

[2. Guide design optimisation 6](#_Toc187156417)

[Cas9 binding buffer optimisation 7](#_Toc187156418)

[3. tracrRNA degree of labelling 9](#_Toc187156419)

[4. qPCR 10](#_Toc187156420)

[5. References 11](#_Toc187156421)

## Image and data processing

Images was converted from .CZI to .tif with Fiji^1^ and subsequently manually inspected for features that potentially could affect the downstream processing. All processing was performed with specialised Python based scripts using implementations from Skimage^2^ and CV2^3^. Data processing and visualisation was done in R^4^  using the ggplot2^5^ package.


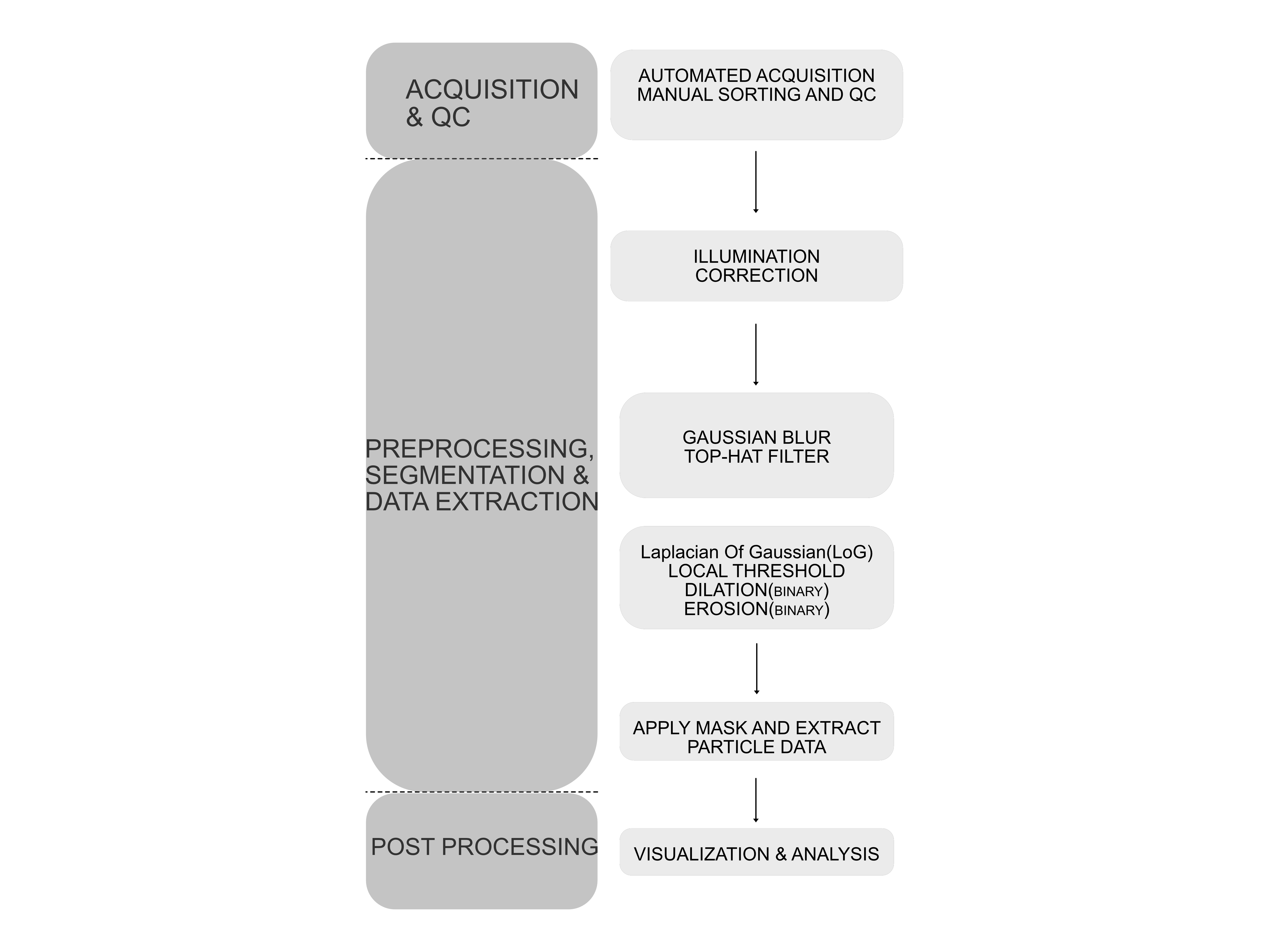


Supplementray Figure 1 Scheme outlining the steps used in image analysis

### Pre-processing

Preprocessing of each image was done in two steps. First step sought to mitigate the variation introduced by uneven illumination. The following steps were implemented to remove noise and improve the precision of the segmentation.

#### Illumination Correction

The uneven illumination profile was corrected for by creating a 2D profile for each experiment from an average of all the images. A 2D gaussian was fitted to the average image which subsequently was used to correct each image(I_i_) according to^6–9^:

$I_{i}^{corr}=\frac{I_{i}}{{2D}_{Gauss}}*mean\left( {2D}_{Gauss} \right)$ (1)

####
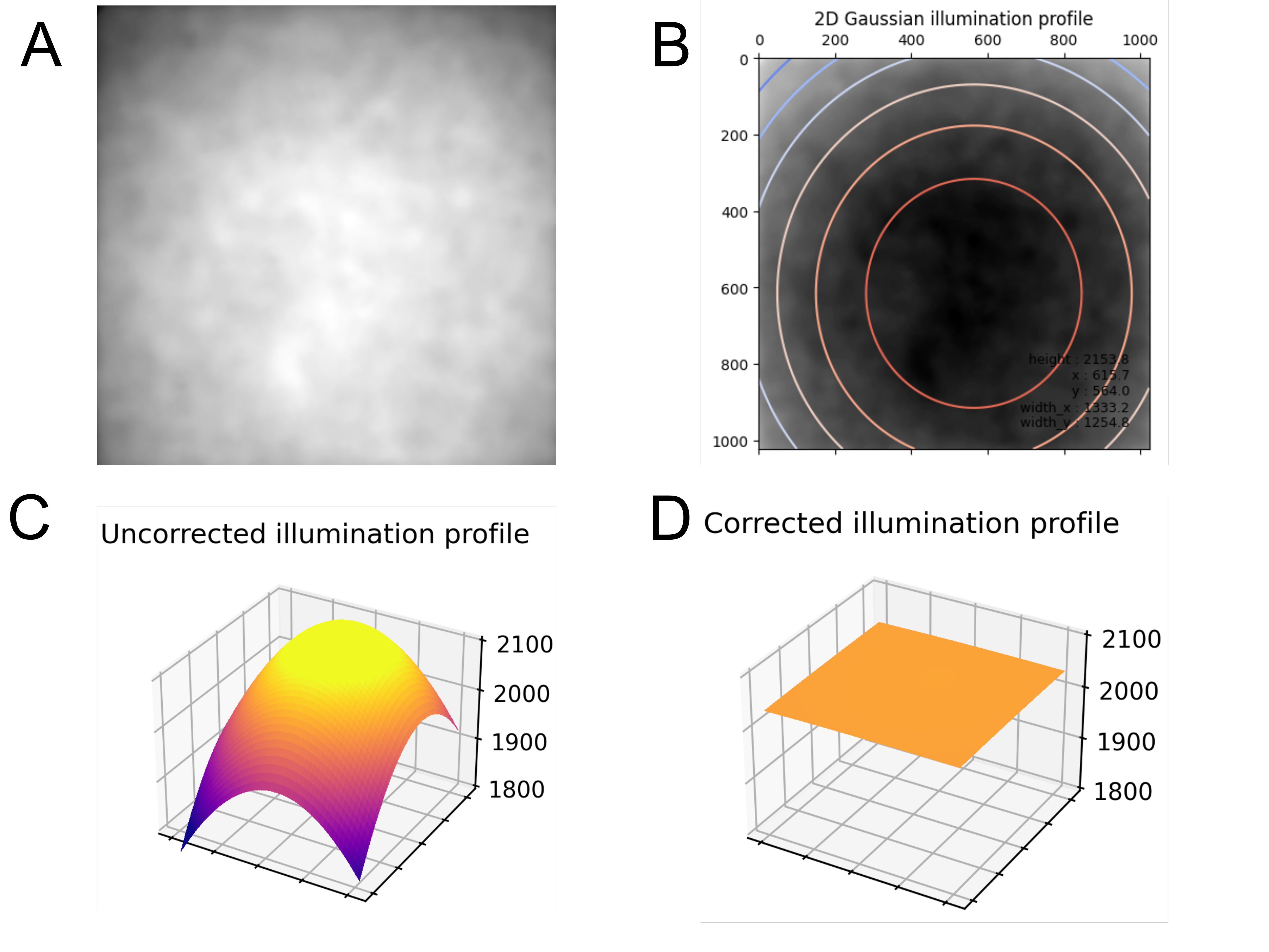


Supplementray Figure 2 **A** Representative mean image used for estimating the illumination profile of each experiment. **B** 2D representation of the gaussian fitted to the mean image. **C** 3D representation of the gaussian fitted to the mean image. **D** 3D representation of the illumination profile after each image have been corrected according to Equation (1) with the gaussian shown in **B** and **C**.

#### Gaussian blur

Before segmentation a gaussian blur was added to each image in order to mitigate noise.

#### Top-hat filter

Each image was convolved with a top-hat filter to remove any larger bright structures and enhance the contrast in the particles of interest

### Segmentation

Segmentation of DNA particles was perfomed by generating a mask based on the overlap between two separate segmentation levels. Laplacian of Gaussian(LoG) and a local thresholding algorithm. LoG was used as an edge-detector to find places in the image with a high and rapid increase in intensity. This gives a first approximation of where the center of a majority of the DNA puncta is located. Since the LoG detection is sensitive to noise and curved edges a second approach is used to mitigate false-positives. The local thresholding algorithm implemented here sets a threshold based on the wheighted mean from a local neighbourhood with a gaussian kernel. Any pixel within the neighbourhood above the mean are considered foreground.

#### Dilation/Erosion

Each binary image was processed by two morphological operations, dilation followed by erosion. Dilation enlarges bright regions and decreases dark regions which essentially removes any holes or very small punctas. Erosion sets a pixel to the minimum in the defined neighbourhood which essentially shrinks all objects as to not overestimate their size and avoid including background pixels.


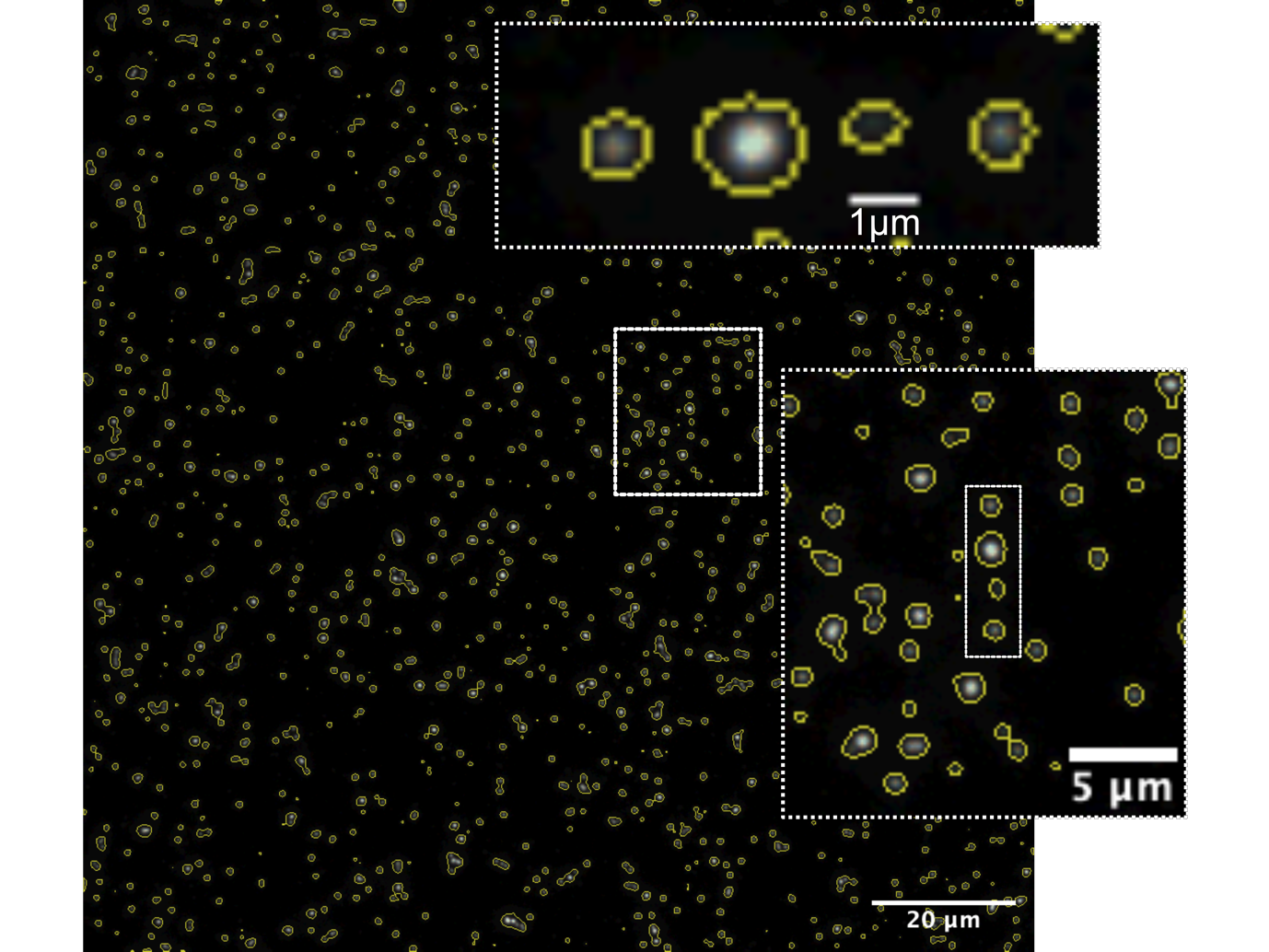


Supplementray Figure 3 Representative output from segmentation of monodispersed plasmid DNA on functionalised glass. Each molecule is defined by the boundaries depicted in yellow.

### Co-localisation statistics

To determine if a DNA-particle was co-localized with a fluorescent object in a separate channel the Pearsons Correlation Coefficient(PCC)^10–12^ was calculated for the foreground pixels between the two channels. The PCC is a measure of the linear correleation between two datasets. A high correlation means that the pixel intensites in both channels are high and thus a indication that there are fluorescence above background in both channels within the segmented area. Low or negative correlation indicates that there are few or no pixels with intensity above background in one of the channels. A DNA puncta was categorised as co-localized if the PCC > 0.25 with a p-value < 0.05.

### Peak identification and Gaussian estimation

To find the intensity of each DNA population each peak center was identified either manually or by 1D k-means clustering^13^ . From the peak center the full width half maximum(FWHM) was extracted and from the FWHM a standard deviation(s) could be estimated:

$FWHM=2\sqrt{2ln(2)}\sigma\approx2.355\sigma$ (2)

The estimated standard deviation(s) and the position of the center(m) was then used to generate an estimated gaussian based on a nonlinear least square(nls^4^ ) fit according to:

$g\left( x \right)=a*e^{{-(\frac{\left( x-\mu\right)}{\sigma})}^{2}}$ (3)

From the estimated gaussian the mean and standard deviation was used to define a subset of data. The center of the subset was then used to fit a new gaussian which was used for further analysis.


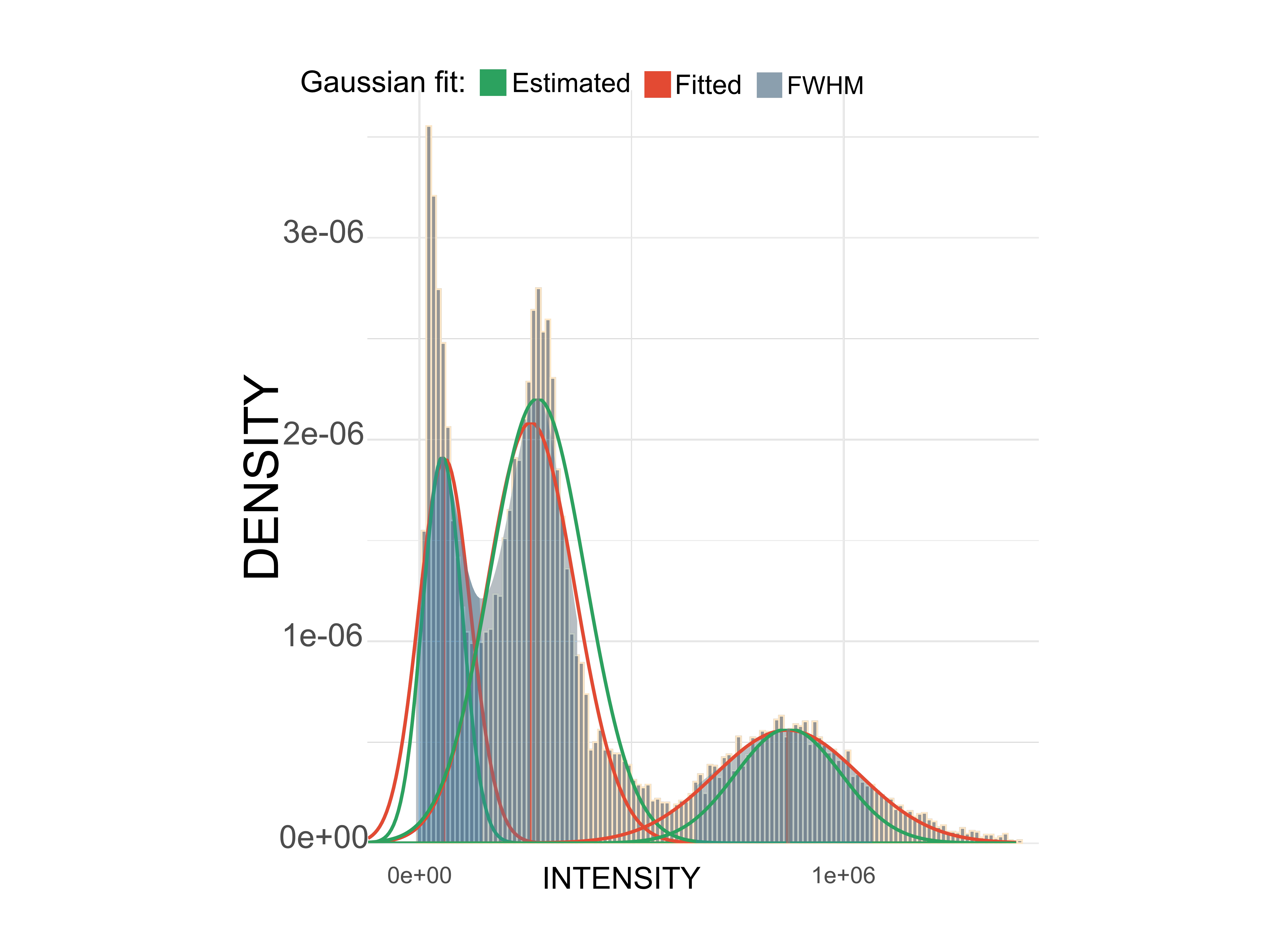


Supplementray Figure 4 Representative example of peaks in a heterogenous plasmid sample with the FWHM, estimated and fitted gaussian mapped out.

1. Guide design optimisation
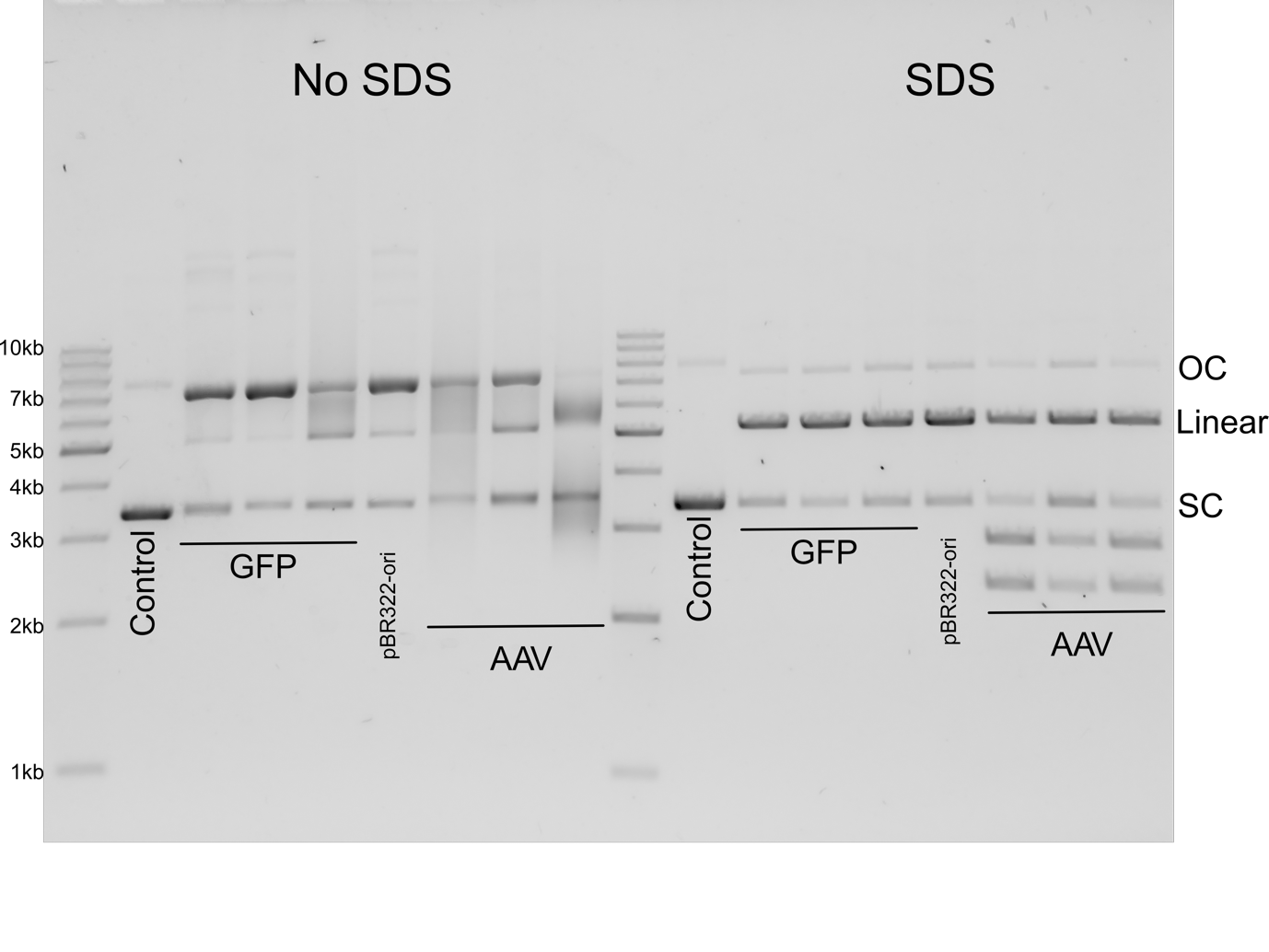


Supplementray Figure 5 Representative gel showing the digestion efficiency by different guide sequences against various targets with a plasmid substrate. The reaction was divided in half and supplementet with loading dye with and without SDS to determine if there was a shift due to the Cas9 that was still bound to the DNA. The image represent one of three independent experiments.


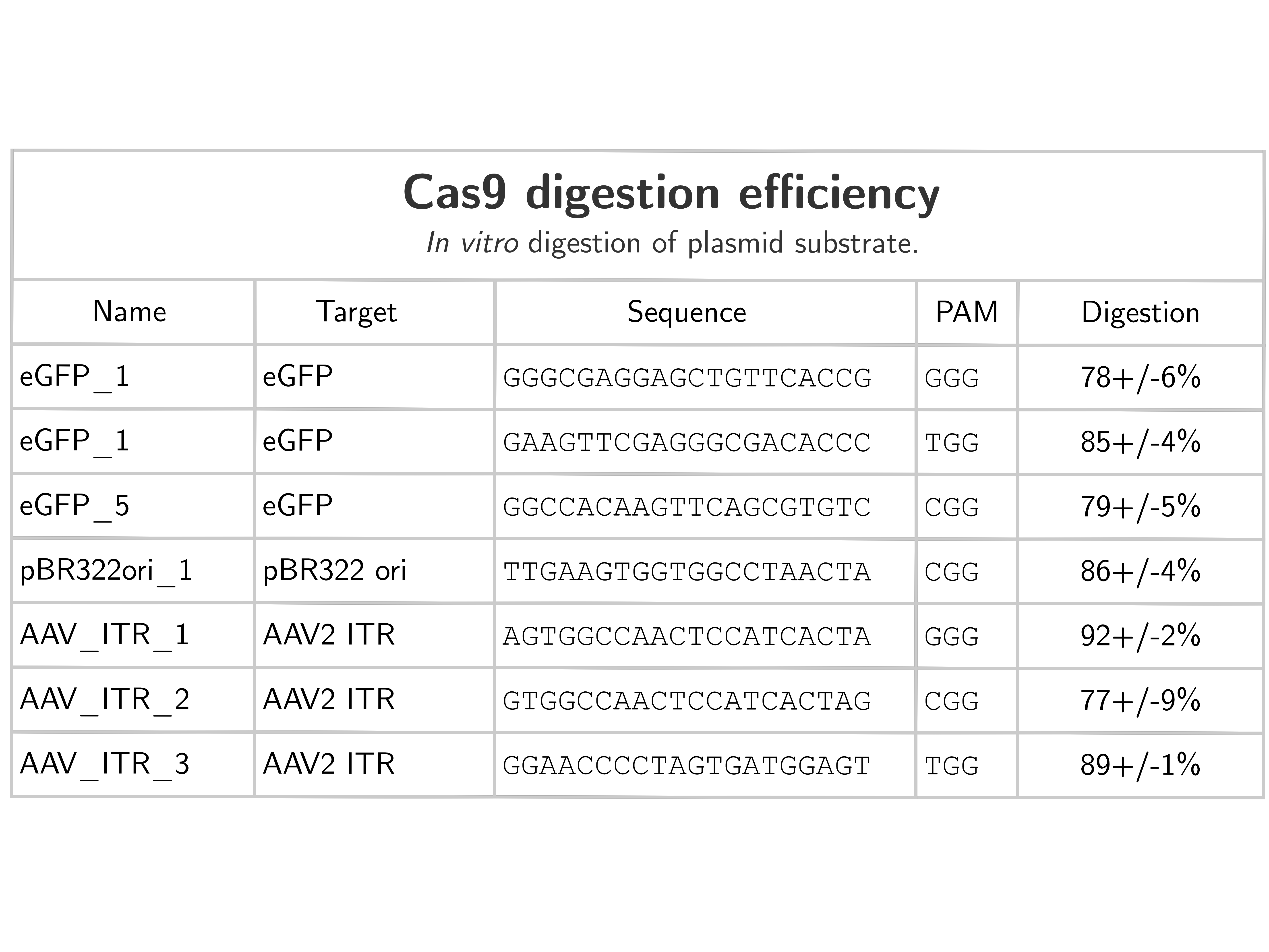


Supplementray Table 1 Table showing the average digestion efficiency by each guide sequence design together with its sequence, PAM and target. The order of the table corresponds to the lanes of the gel shown in Supplementary Figure 4.

## Cas9 binding buffer optimisation

It has previously been observed that the presence of MgCl_2_ can interfere with the binding of YOYO-1 to DNA^41^. Since the function of Cas9 is dependent on MgCl_2_^42^, digestion experiments were done to determine a lower limit of MgCl_2_ where the nucleolytic function was still retained. The digestion was sufficiently efficient at the low end of the gradient (1-10 mM) with 94.9+/-0.7% digestion efficiency at 1mM of MgCl_2_ (**Supplementary Figure** **6A**). Similarly, it has been demonstrated that YOYO-1 can interfere with enzymatic reactions^41,43^. The effect of YOYO-1 on dCas9 binding was investigated with an EMSA with varying amounts of bp:YOYO-1 ratios (**Supplementary Figure** **6B)**. The results show that dCas9 is inhibited by excessive amounts of YOYO-1 in the reaction (1:1). A moderate excess (3:1) causes a shift although smeary which can be interpreted as unspecific binding. A ratio of 5:1 and below gives a discernable shift with distinct bands. Based on this it is apparent that any concentration of YOYO-1 above 5:1 will interfere with the binding of dCas9 to DNA. These results were confirmed by investigating the digestion efficiency of a nucleolytically active Cas9 in the same bp:YOYO-1 ratios (data not shown). To achieve sufficient and replicable signal strength, a bp:YOYO-1 ratio of 9:1 was chosen as a compromise based on observations from microscopy experiments. Since the efficiency of dCas9 is also dependent on target:dCas9 ratio the binding of dCas9 was confirmed over a range of increasing target:dCas9 ratios, **Supplementary Figure** **6C**. The EMSA shows that the binding plateaus at a ratio above 15.

The assumption that YOYO-1 could interfere with the binding of dCas9 to DNA was somewhat confirmed by the EMSA results, but not to the degree anticipated. When doing a densiometric comparison between lanes with and without dCas9 a small decrease (~16%+/-1) in lanes with dCas9 was observed. This raised the question whether there might be a reversed problem where dCas9 interfered with the binding of YOYO-1. To investigate this, the DNA was either labelled before the dCas9 was added or after. Simultaneously the specificity of the dCas9-RNA complex was confirmed by the inclusion of a plasmid lacking a dCas9 binding site, **Supplementary Figure 6D**. The results indicate that dCas9 binds DNA equally well, regardless of when the YOYO-1 is introduced. Interestingly, the order in which YOYO-1 and dCas9 is added to the reaction seems to influence the binding of YOYO-1 to DNA regardless of whether the DNA contains a dCas9 binding site or not. Of note,is that there is a small shift when using DNA without a target site, suggesting that dCas9 could have unspecific DNA binding activity under the specific conditions used. Based on these results all consecutive experiments including dCas9 was performed so that YOYO-1 was introduced to the DNA prior to the addition of dCas9.


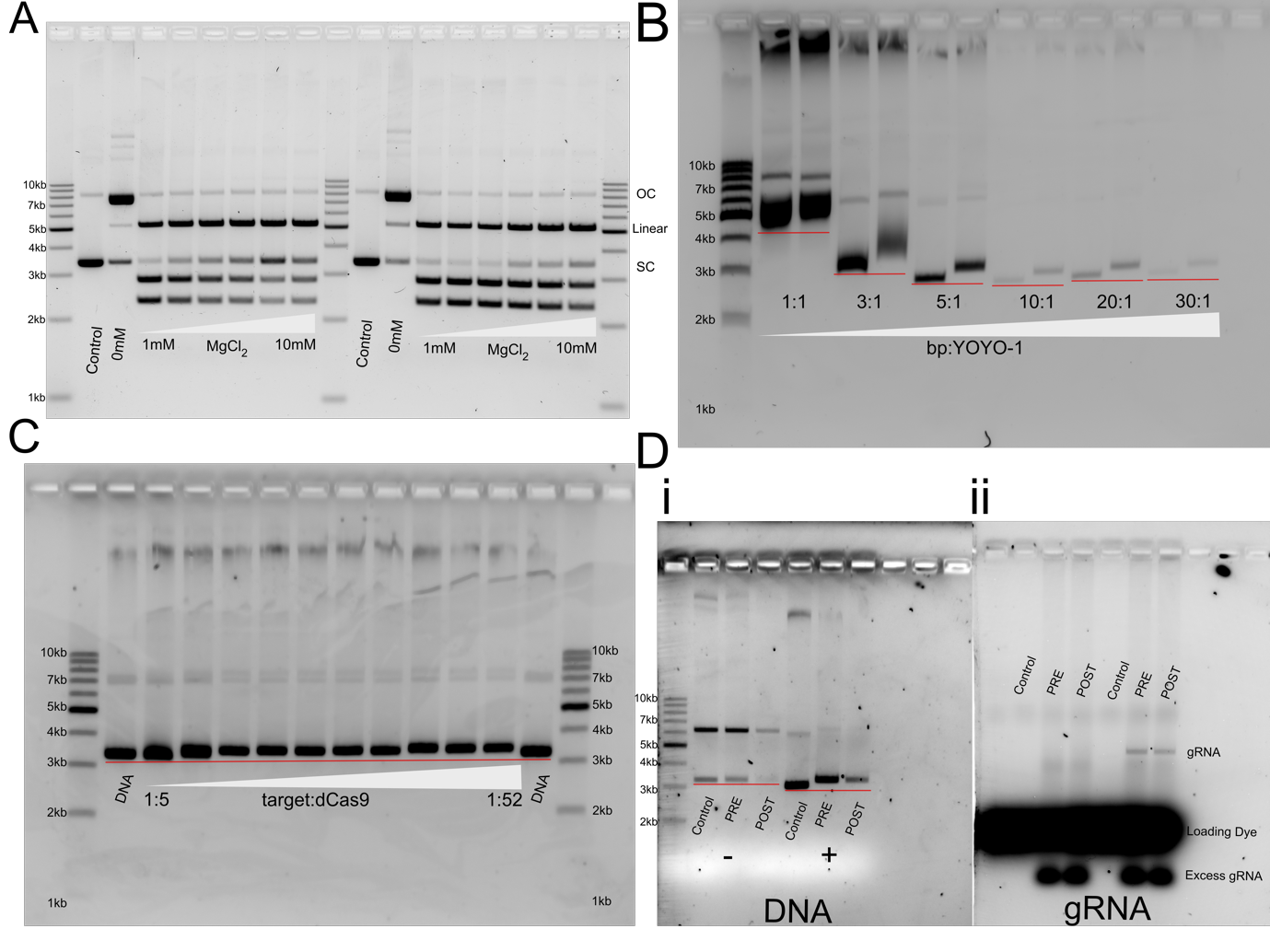


Supplementray Figure 6 **A** Two replicates of Cas9 digestion reactions with MgCl_2_ gradients to determine the Cas9 digestion efficiency dependence on MgCl_2_. Reaction was done in NEBuffer r3.1 with varying concentrations of MgCl_2_ **B** EMSA with dCas9(NEB EnGen® Spy dCas9 (SNAP-tag®)) to establish the effect of YOYO-1 on the binding of dCas9 to plasmid DNA. Reaction was done in NEBuffer r3.1 with 1mM MgCl2 and 1mM DTT **C** EMSA experiment conditions was 9:1 bp:YOYO-1 with a decreasing ratio of target:RNP. YOYO was added prior to the Cas9-RNA complex and incubated in 50C for 1hr before the Cas9-RNA complex was added and incubated 15 min. Reaction was done in NEBuffer r3.1 with 1mM MgCl2 and 1mM DTT **D** EMSA with dCas9(NEB EnGen® Spy dCas9 (SNAP-tag®)) to determine the effect of dCas9 on the binding of YOYO-1 to DNA. “-“ and “+” indicates if the DNA substrate used contained a target site for the Cas9 to bind. “Control” refers to reactions without dCas9 or RNA included. “PRE” and “POST” indicates whether the DNA was incubated before or after the addition of dCas9. Reaction was done in NEBuffer r3.1 with 1mM MgCl2. **i)** Image from YOYO-1 specific exposure **ii)** Image from ATTO550(fluorophore on RNA) specific exposure.

# tracrRNA degree of labelling


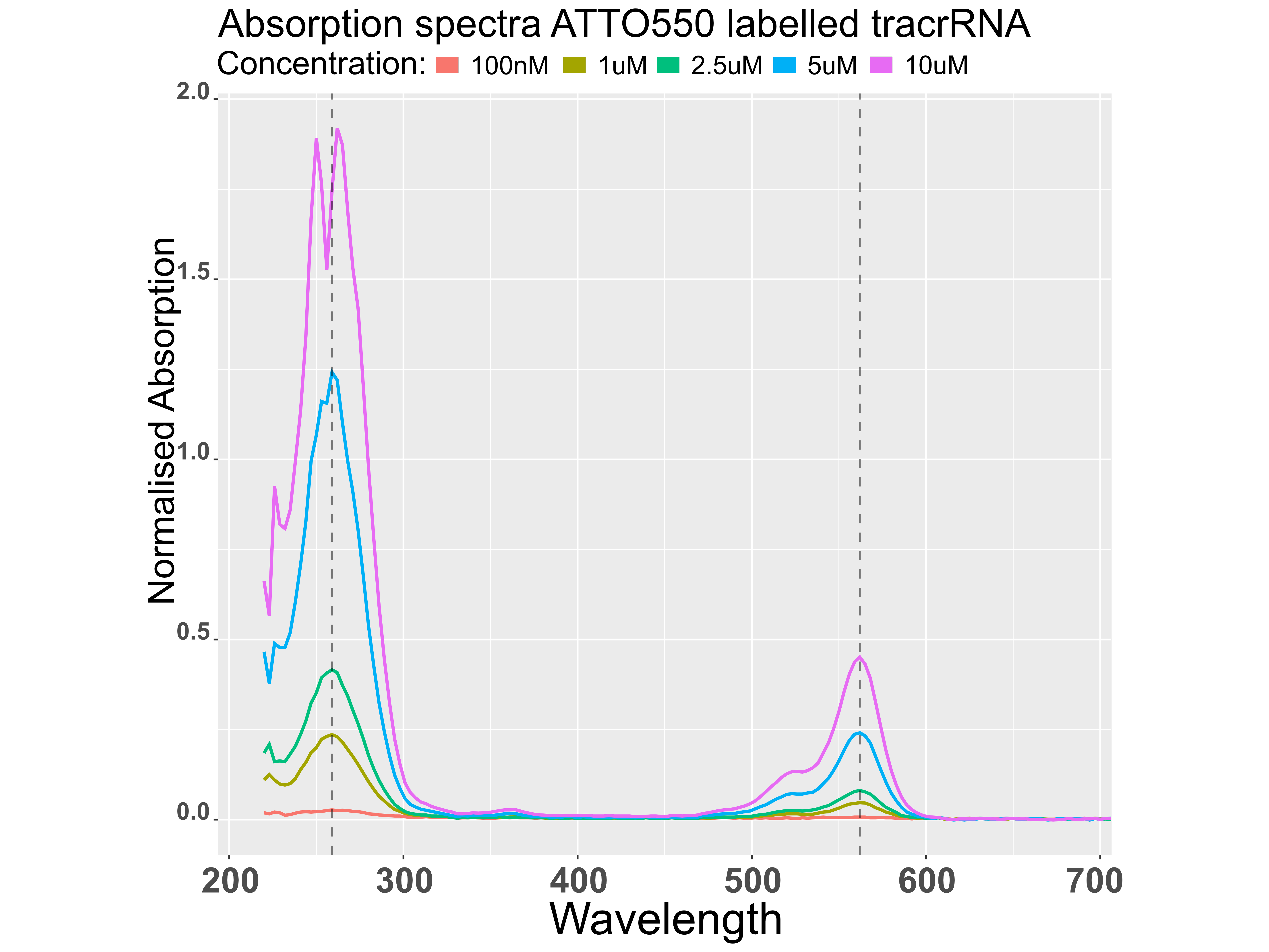


Supplementray Figure 7 UV/Vis absorption spectra of Alt-R CRISPR-Cas9 tracrRNA - ATTO™ 550 with absorption maximums, 260nm and 558nm, marked with dashed lines.


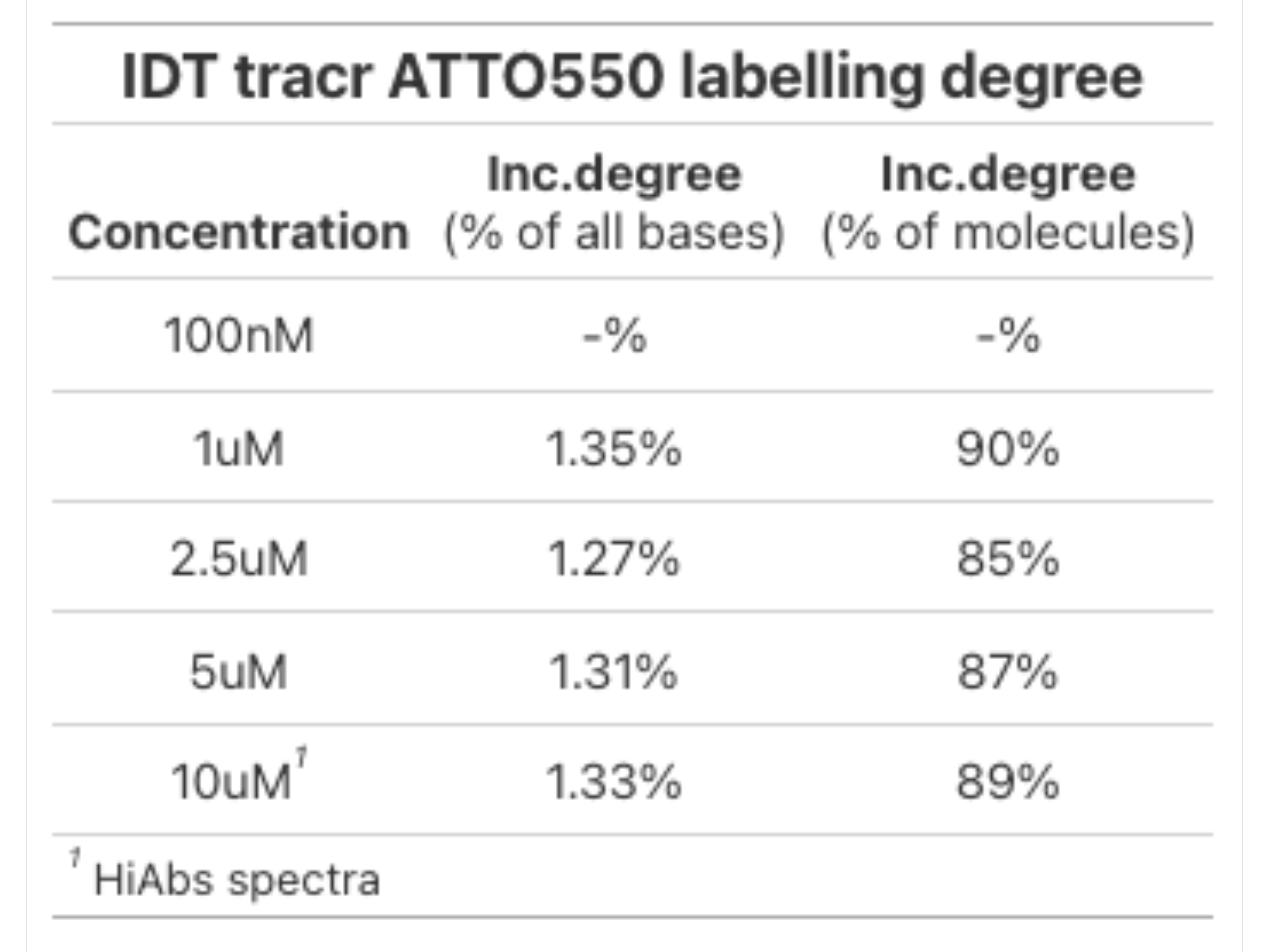


Supplementray Table 2 Calculated degree of labelling(DoL) of Alt-R CRISPR-Cas9 tracrRNA - ATTO™ 550 at different concentrations based on the data from Supplementray Figure 5. The mean DoL is 87.75%.

# qPCR


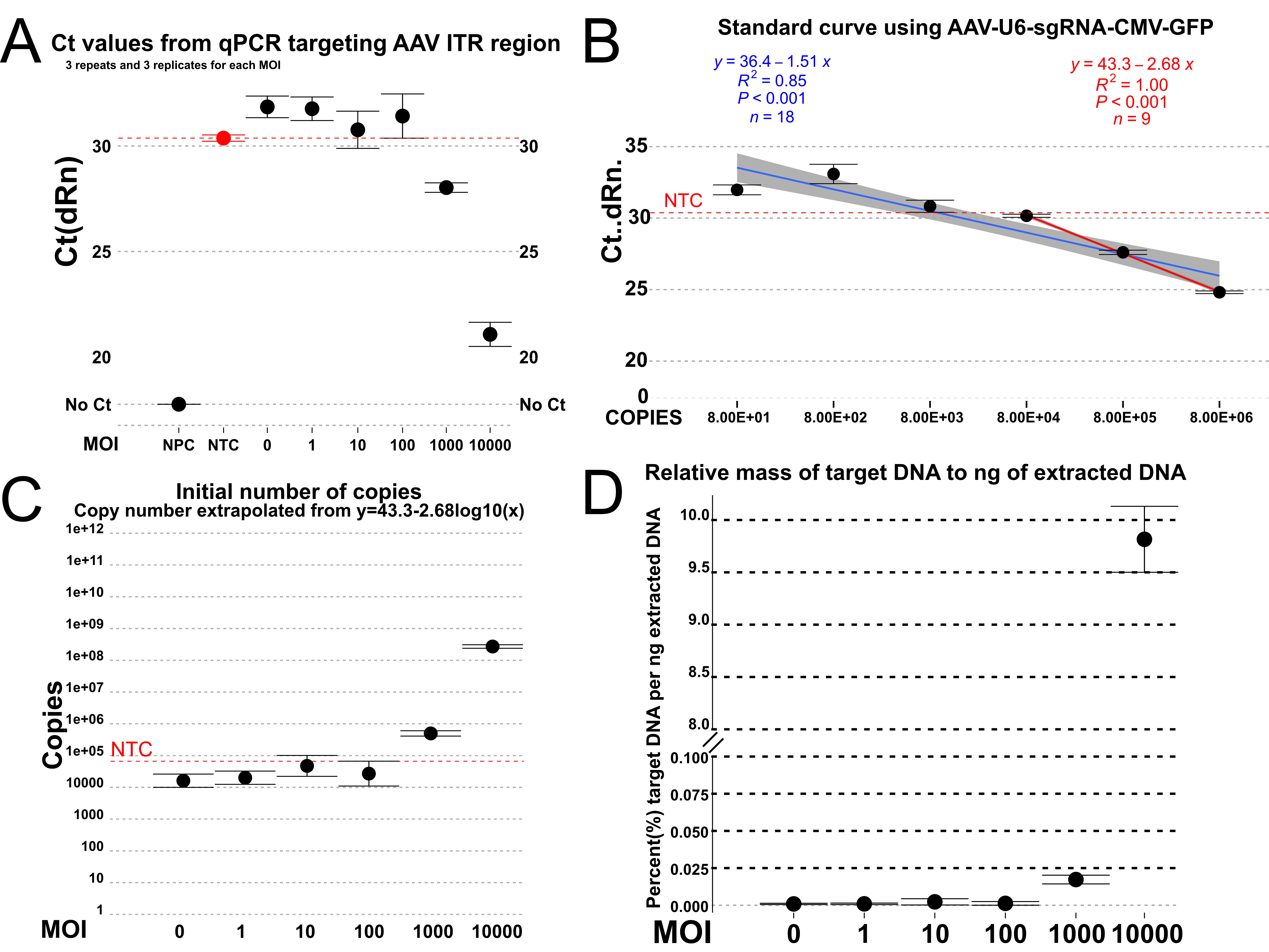


Supplementray Figure 8 **A** Average Ct values from 6 technical replicates with the non-primer control(NPC) and non-template control(NTC) as references. **B** Standard curve for mapping of Ct values and initial number of copies in a qPCR reaction. **C** Copy numbers for each condition after mapping of Ct values from **A** via equation from **B.** NTC level marked with a dahed red line. **D** Relative mass of viral vector in relation to mass of extracted DNA.

# References

1. Schindelin, J. *et al.* Fiji: an open-source platform for biological-image analysis. *Nat Methods* **9**, 676–682 (2012).

2. Walt, S. van der *et al.* scikit-image: image processing in Python. *PeerJ* **2**, e453 (2014).

3. Bradski & G. The OpenCV Library. *Dr. Dobb’s Journal of Software Tools* (2000).

4. Team, R. C. R: A Language and Environment for Statistical Computing. (2024).

5. Wickham, H. ggplot2: Elegant Graphics for Data Analysis. (2016).

6. Wolf, D. E., Samarasekera, C. & Swedlow, J. R. Chapter 14 Quantitative Analysis of Digital Microscope Images. *Methods Cell Biol.* **114**, 337–367 (2013).

7. Model, M. Intensity Calibration and Flat‐Field Correction for Fluorescence Microscopes. *Curr. Protoc. Cytom.* **68**, 10.14.1-10.14.10 (2014).

8. Jost, A. P.-T. & Waters, J. C. Designing a rigorous microscopy experiment: Validating methods and avoiding bias. *J. Cell Biol.* **218**, 1452–1466 (2019).

9. Young, I. T. Shading Correction: Compensation for Illumination and Sensor Inhomogeneities. *Curr. Protoc. Cytom.* **14**, 2.11.1-2.11.12 (2000).

10. Dunn, K. W., Kamocka, M. M. & McDonald, J. H. A practical guide to evaluating colocalization in biological microscopy. *Am. J. Physiol.-Cell Physiol.* **300**, C723–C742 (2011).

11. BOLTE, S. & CORDELIÈRES, F. P. A guided tour into subcellular colocalization analysis in light microscopy. *J. Microsc.* **224**, 213–232 (2006).

12. Costes, S. V. *et al.* Automatic and Quantitative Measurement of Protein-Protein Colocalization in Live Cells. *Biophys. J.* **86**, 3993–4003 (2004).

13. Wang, H. & Song, M. Ckmeans.1d.dp: Optimal k-means Clustering in One Dimension by Dynamic Programming. *R J.* **3**, 29–33 (2011).
